# Does focused attention meditation improve probability weighting bias? The role of emotional trade-off difficulty and compensatory decision making

**DOI:** 10.1186/s40359-026-04053-z

**Published:** 2026-02-03

**Authors:** Chuyu Zhi, Biqin Li

**Affiliations:** School of Psychology, Jiangxi Normal University, Nanchang, 330022 China

**Keywords:** Emotional trade-off difficulties, Probability weighting bias, Mindfulness, Compensatory decision making, Meditation, Focused attention

## Abstract

**Background:**

Focused attention (FA) meditation has been proposed to enhance decision quality by mitigating cognitive biases. However, its influence on probability weighting bias (PWB), the tendency to misperceive objective probabilities, remains unexplored. Furthermore, emotional trade-off difficulty (ETOD), defined as the affective strain of making complex trade offs, may influence this relationship. The present study explores the interplay between FA meditation, ETOD, and PWB. Specifically, we examined whether ETOD’s effect on PWB is mediated by compensatory decision strategies (systematic trade-off evaluation versus reliance on heuristics), and whether a brief FA induction moderates this process.

**Methods:**

A 2 × 2 between-subjects experiment was conducted with 138 participants randomly assigned to either a brief focused attention or a mind-wandering control condition. Participants completed a decision-making task to induce either high or low levels of ETOD. PWB was assessed through a series of risky-choice scenarios, while gaze-time tracking measured the extent of compensatory information processing, proposed as a mediating variable.

**Results:**

Under high ETOD, participants who underwent FA induction demonstrated significantly greater PWB compared to those in the control group, suggesting that FA meditation exacerbated decision bias under emotional strain. In contrast, under low ETOD, FA meditation reduced PWB. Moderated mediation analysis revealed that ETOD influenced PWB through its impact on compensatory decision strategies, with this pathway being moderated by FA induction.

**Conclusions:**

Our results demonstrate that FA meditation effectively mitigates PWB under low ETOD conditions but does not yield the same benefit under high ETOD. Consequently, we conclude that the utility of FA meditation is strictly context-dependent.

## Background

The concept of probability weighting bias (PWB) refers to the phenomenon where subjective estimates of how likely an event is to occur (the weight of probability) deviate from the event’s actual probability [1–3]. Typically, the bias is represented by an inverse S-shaped probability weighting function (PWF), indicating that individuals overweight small probabilities and underweight large probabilities [1, 4, 5]. Such distortions in probability judgments can have significant effects in real-world scenarios. For example, during the COVID-19 pandemic, some individuals underestimated the risk of infection and refused to wear masks, thereby obstructing public health initiatives. Mindfulness, defined as the purposeful and non-judgmental focusing of attention on the present moment, has recently garnered considerable interest as a potential means of enhancing decision making. Mindfulness induction refers to a single session of brief meditative practices, typically lasting between 5 and 45 min [6]. It often involves the utilization of FA meditation [7], OM meditation [8], or a combination of both [9]. The FA meditation (FA) used in this study entails sustaining attention toward an anchor object (e.g., the sensation of breathing), maintaining self-awareness when the mind wanders, and directing attention back to the anchor object [10]. Given that PWB often stems from an imbalance in processing abstract probability versus concrete outcome information due to limited cognitive capacity [5, 11], the potential of FA to enhance top-down attentional control [12] suggests a theoretical link for mitigating this bias. Whether mindfulness can enhance decision quality, particularly in the context of biases like PWB, is a question of substantial practical importance. The present study aims to provide a nuanced understanding of the intricate relationship between FA meditation induced mindfulness and PWB.

The potential for mindfulness to modulate decision making is supported by neurobiological evidence suggesting overlapping functional pathways shared by these two processes. Decision making is shaped by distinct neural pathways [13]. Emotional decisions tend to increase activity in the medial prefrontal and medial orbitofrontal cortices, as well as their striatal counterparts. In contrast, more rational or deliberative decisions are typically associated with increased activity in the lateral prefrontal and parietal regions [14, 15]. Neuroimaging evidence indicates that even brief mindfulness training can alter neural activity in regions involved in social decision making, such as the medial prefrontal cortex [16]. Mindfulness practice is further associated with enhanced relaxation-related activity in the anterior cingulate cortex, an area involved in regulating attention and emotions [17]. Meditation also activates the insular cortex, which is central to interoceptive awareness, or sensing internal bodily states. Voxel-based morphometry studies reveal that experienced meditators possess greater gray matter volume in the insula compared to non-meditators, suggesting that long-term mindfulness practice can lead to structural changes in the brain [18, 19]. These findings collectively suggest that mindfulness training primarily influences brain regions associated with emotion and intuition. Similarly, the Mindful Coping Model highlights the role of mindfulness in regulating emotions, promoting positive reappraisal, and improving strategies for managing emotional responses [20].

PWB is commonly assessed using decision-making tasks that engage the prefrontal and parietal cortices, which are key regions involved in evaluating risk and processing complex cognitive information [21]. In turn, mindfulness may facilitate emotional regulation by influencing neural circuits associated with the emotional system and by altering the allocation of attentional resources during decision making. In the decision-making literature, PWB is linked to the use of compensatory versus non-compensatory strategies. As individuals have limited capacity to process sensory information, they frequently rely on heuristic judgments rather than considering all available evidence [11, 22]. Outcome information is usually concrete and vivid, making it easier to process, whereas probability information is more abstract and less intuitive [5, 11, 23]. This tendency predisposes individuals to adopt non-compensatory strategies, placing greater emphasis on outcomes rather than probabilities, which increases the likelihood of PWB [11, 24]. In contrast, compensatory strategies involve more balanced consideration of both outcome and probability information and are associated with reduced bias. Building on this, research has found that mindfulness practice increases functional connectivity between the frontoparietal network and the default mode network, suggesting enhanced top-down attentional control and better regulation of mind wandering [12]. Given the established benefits of mindfulness for sustaining attention, FA meditation may help individuals allocate more balanced attentional resources between probability and outcome information. By improving attention control, FA meditation could promote the use of compensatory strategies in decision making, thereby reducing PWB.

While mindfulness may enhance decision making, likely through emotional pathways, previous research has not fully considered the effects of emotional trade-off difficulty (ETOD) within the decision-making process. In the context of anticipatory emotions, ETOD refers to the experience of negative affective states, such as guilt, conflict, or distress, that arise when individuals weigh attributes connected to valued goals, including life preservation, health outcomes, environmental sustainability, or time constraints [25, 26]. These emotionally laden challenges can impair the capacity for rationally evaluating competing priorities. It is important to distinguish between task-related emotions, such as ETOD, and task-unrelated emotions, such as those arising from environmental contexts. Although the mood literature does not offer a coherent hypothesis about the effects of negative affect on decision processing, it consistently links higher levels of negative emotion with more extensive and alternative-based cognitive processing [27–30]. In decision-making situations, negative emotions that are directly tied to the task may prompt more extensive and attribute-focused processing, often as an attempt to avoid making difficult trade-offs [31, 32]. This view aligns with arguments that the impact of emotions on cognitive processing depends, in part, on how closely those emotions are related to the task itself [33]. Therefore, elucidating the influence of FA meditation on decision making necessitates a concurrent investigation into the impact of ETOD on these psychological processes.

Most research on ETOD has focused on the decision-making process itself. Studies have shown that as individuals move from low to high levels of ETOD, they often shift from attribute-based processing to alternative-based processing, a transition typically accompanied by longer search times and more extensive information gathering [31, 34, 35]. However, the negative emotion hypothesis alone offers an incomplete explanation for the outcomes of these psychological processes, thereby limiting the predictability of emotional influences on PWB. The issue of selectivity in decision making may help to explain how ETOD predicts PWB. Just as mindfulness may influence the use of compensatory strategies, ETOD may also shape compensatory strategies and, in turn, affect PWB. The effort-accuracy model suggests that decision making involves selecting strategies based on the need for accuracy in a given context and the cognitive resources available [25]. Within this framework, the negative emotion hypothesis examines how negative emotions can influence decision making by changing either the decision-maker’s accuracy requirements or the cognitive resources at their disposal. As negative emotions signal the importance of a decision, it motivates individuals to prioritize accuracy and encourage analytic, alternative-based processing that is characterized by more extensive, less selective information search [36]. On the other hand, negative emotions can disrupt attentional focus and reduce cognitive resources, leading individuals to rely on heuristic processing strategies that are less extensive, more selective, and attribute-based [30]. Selectivity in decision making refers to the extent to which information is processed selectively, whether different amounts of information are considered for various attributes or alternatives, or consistently, where the same amount of information is processed for all options [31]. Compensatory decision strategies involve making explicit trade-offs between attributes, such as sacrificing salary for better career opportunities for a spouse, and are marked by reduced selectivity [31, 37]. On the other hand, non-compensatory decision rules are often preferred because they allow individuals to avoid the negative emotions associated with making difficult trade-offs [38]. These non-compensatory decisions, characterized by high selectivity, are typically associated with attribute-based processing and reflect an avoidance-driven approach to decision making.

This study proposes that the two opposing effects of negative emotions on decision making operate at different levels of emotional trade-off difficulty, depending on the availability of cognitive resources. At low levels of ETOD, negative emotions do not significantly distract attention, allowing individuals to retain sufficient cognitive resources for thorough information processing. In this context, negative emotions act as a motivating factor, encouraging alternative-based processing and reducing selectivity, which is reflected in an increase in compensatory decision making. In contrast, at high levels of ETOD, negative emotions draw heavily on cognitive resources, leading to attention deficits and insufficient capacity for comprehensive information processing. Under these circumstances, individuals are more likely to engage in attribute-based processing and adopt non-compensatory decision strategies. Notably, while negative emotions at high ETOD can increase search time and information gathering owing to their motivational effect, the consequent cognitive overload still leads to more selective and avoidance-driven decision patterns. Consequently, increasing levels of ETOD are likely to intensify PWB by influencing the degree of compensatory processing.

Previous research examining the impact of emotion on PWB has shown that negative emotions induced by high-intensity emotional states produce greater bias than those induced by lower-intensity states, even when trade-off considerations are not explicitly included [39]. FA meditation has the potential to alleviate the negative emotions produced by ETOD. Existing studies demonstrate that mindfulness enhances emotional regulation, as evidenced by increased activity in the anterior cingulate cortex, which is linked to improved emotional control [17]. Mindfulness interventions have also been shown to reduce stress and negative emotions in practical settings [40, 41]. Research further suggests that mindfulness can improve decision-making quality [42]. Since ETOD inherently generates negative emotions by requiring individuals to weigh conflicting options, FA meditation may help mitigate these emotional difficulties. Given its documented benefits on emotional regulation and decision making, this study suggests that FA meditation may reduce the negative emotional impact of ETOD. By doing so, FA meditation could influence how individuals allocate attention to probability and outcome information, which in turn would affect selectivity and compensatory decision making, ultimately decreasing PWB and optimizing decision outcomes.

We propose a unified framework grounded in Bishop et al.‘s two-component model of mindfulness [43], adopting an Attentional Regulation Perspective to elucidate how ETOD and FA meditation jointly shape decision-making quality (i.e., Probability Weighting Bias, PWB). According to this model, mindfulness comprises two distinct components: (1) the self-regulation of attention, which involves sustaining focus on immediate experience and inhibiting elaborative processing; and (2) a specific orientation to experience, characterized by curiosity, openness, and acceptance. Given our focus on cognitive resource allocation and the Focused Attention (FA) practice, our framework explicitly prioritizes the first component—the self-regulation of attention—as the central mechanism. However, we posit that the second component (orientation to experience) serves a critical facilitating role. Specifically, a non-judgmental orientation attenuates the initial emotional reactivity to ETOD, thereby limiting the generation of elaborative processing at the source. This supportive function reduces the regulatory burden on the first component, enabling the primary mechanism—attentional regulation—to inhibit intrusive thoughts more efficiently with fewer cognitive resources. Within this framework, rational, compensatory processing (the mediator) is fundamentally constrained by attentional control: it relies on the self-regulation of attention [44]—the first component of Bishop’s model—to allocate limited cognitive resources toward analytical tasks. Drawing on the Resource Allocation Model [45], which posits that mood states interfere with the allocation of cognitive resources, and building on the insight derived from the Negative Emotion Hypothesis regarding the heightened consumption of cognitive resources under high ETOD, we posit that ETOD imposes a challenge to this cognitive system. At low levels, ETOD may serve as a motivational cue [36]; however, as ETOD intensifies, it triggers severe negative affect that captures attention [30], initiating what Bishop terms “elaborative processing”. This maladaptive secondary processing drains the cognitive resources required for compensatory decision making, forcing the cognitive system to default to resource-sparing heuristic strategies (Non-compensatory decision strategies), thereby increasing PWB (Hypothesis 1). Mindfulness, induced via FA meditation, acts as a critical moderator in this dynamic. Specifically, FA meditation explicitly trains the capacity for inhibition of elaborative processing. Leveraging this attentional regulatory capacity, FA meditation buffers the detrimental impact of ETOD on compensatory decision making. By detecting and inhibiting the elaborative processing triggered by ETOD, FA meditation prevents the cognitive resource depletion that typically disrupts this analytical process. This preservation of cognitive resources allows individuals to sustain compensatory processing—simultaneously trading off attributes and probability weights—even under conditions of emotional load. Consequently, by liberating cognitive resources from such elaboration, FA meditation attenuates the intensity of the negative relationship between both high and low levels of ETOD and compensatory processing, thereby mitigating PWB. In this way, induction moderates the indirect effect of ETOD on PWB via compensatory processing (Hypothesis 2). However, it is important to acknowledge that this moderating effect operates within the constraints of limited cognitive resources. Since the act of self-regulating attention in FA involves enhanced top-down regulation—evidenced by increased functional connectivity between the frontoparietal network and default mode networks during this practice [12]—it represents an active process that inherently consumes cognitive resources. Consequently, the net benefit of FA meditation depends on the balance between resources conserved (via inhibition) and resources invested (via regulation). Therefore, while FA meditation is generally expected to facilitate compensatory processing and mitigate PWB, this positive moderation is contingent upon the availability of sufficient cognitive resources to support both the regulatory effort and the decision task.

This study introduces several new contributions to the literature. While previous research has explored the influence of emotions on the probability weighting function, it often overlooks the emotional dynamics generated by trade-offs. For instance, studies on probability assessments and emotional arousal frequently present participants with choices between two aversive options, such as a monetary fine or a brief electrical shock [39]. Although these scenarios involve trade-offs, the resulting negative emotions are often neglected. Trade-offs are common in decision making, especially in contexts where emotions and PWB are studied, and accurately predicting probability assessments requires isolating and addressing the influence of trade-off-related emotions. Another contribution of this study is its examination of the tension between two common theoretical explanations for ETOD. Neither the negative emotion hypothesis nor the coping behavior framework fully accounts for observed findings, particularly with regard to selectivity, which remains inadequately explained by either approach. By offering a new perspective, this study addresses the selectivity issue and provides fresh insights into ETOD research. Finally, this study explores FA meditation as a potential strategy for regulating the negative emotions associated with ETOD. While previous research on mindfulness and decision making has largely focused on general improvements in decision quality, this study highlights the importance of considering ETOD. Some studies suggest that cognitive control may serve as a theoretical foundation for understanding the unified effects of mindfulness on decision making, though further research is required [46]. This study investigates whether FA meditation can influence the mechanisms that mediate the relationship between ETOD and PWB, clarifying the cognitive and psychological processes involved in trade-offs and providing a new perspective on the relationship among FA meditation, emotion, and decision making in complex situations.

Based on the above, this article proposes the following hypotheses:

- H1: ETOD negatively affects compensatory processing, which in turn increases PWB.
- H2: Induction moderates the indirect effect of ETOD on PWB via compensatory processing.

## Methods

### Study design

This experimental study employed a 2 × 2 between-subjects design. Randomization sequentially assigned participants to of four conditions: induction (focused attention vs. mind-wandering) and ETOD (high vs. low). The study received ethical approval from the ethical review committee at the first author’s institution. All participants provided written informed consent and were informed of their right to withdraw at any time without any negative consequences.

### Participants

An a priori power analysis was conducted using G*Power 3.1 to determine the required sample size. Effect sizes were derived from previous research, which typically reported medium to large effect sizes of ETOD on PWF [47, 48]. Adopting a conservative approach, a medium effect size (*d* = 0.50) was specified, resulting in a calculated sample size requirement of 128 participants. The final sample consisted of 138 participants.

Participants were recruited using convenience sampling by approaching student groups at a university location. Eligible students were invited to participate based on the following inclusion criteria: they were required to be college students, proficient in written Chinese, and free of affective disorders, as such conditions could interfere with the decision-making process [4, 49].

To screen for probable depression, the Chinese version of the Patient Health Questionnaire-2 (PHQ-2) was utilized [50]. The PHQ-2 assesses depressive symptoms over a two-week recall period with two items, rated on a 4-point Likert scale (0 = “not at all” to 3 = “nearly every day”). The total score ranges from 0 to 6, with higher scores reflecting greater severity of depressive symptoms. A cutoff score of 3 was applied [51]. Similarly, to screen for anxiety-related disorders, the Chinese version of the Generalized Anxiety Disorder-2 (GAD-2) was used [52]. Similar to the PHQ-2, the GAD-2 assesses symptoms over a two-week recall period and comprises two items rated on the same 4-point Likert scale. The total score also ranges from 0 to 6, and a cutoff score of 3 was adopted for identifying probable cases of anxiety [53, 54].

## Materials and procedures

To ensure that all participants adequately understood the nature of the induction task, we implemented the following steps. First, the audio induction scripts were piloted with a separate sample of six individuals to assess clarity, pacing, and ease of understanding; their feedback confirmed that the instructions were unambiguous and easily comprehensible. Second, in the main experiment, prior to starting the induction, participants received a standardized introductory briefing to ensure they were prepared to engage with the audio guide. Following the completion of the induction, all participants were asked to report on their understanding of the instructions; no participant reported any difficulty in comprehending the script.

For the induction, participants were assigned to either a FA group or a mind-wandering group as a control. Both inductions were delivered via approximately 11 min of audio recordings narrated by a female mindfulness instructor. The FA induction consisted of a mindful breathing exercise, instructing participants to focus on their breathing and non-judgmentally return their attention to the present moment if distracted. The mind-wandering condition is commonly employed as an active control in mindfulness experimental studies [55]. Mind wandering, defined as the occurrence of spontaneous, task-unrelated thoughts and conceptually opposite to FA [56], was induced by instructing participants to freely allow their thoughts to flow, with an emphasis on creativity and unrestricted mental wandering. The audio provided only minimal guidance and included extended silent segments, enabling participants to engage in thinking, daydreaming, or fantasizing without employing goal-directed attentional control. Crucially, this procedure omitted training in the core aspects of FA: concentrated attention, an awareness of mind wandering, and a non-judgmental attitude. Both the FA and mind-wandering induction scripts were adapted from a previous study investigating the effects of mindfulness inductions on memory [57].

After completing the induction, participants performed a charity support task. The task was programmed using the R software with the Shiny package, which resembles the MouselabWEB application for assessing decision making that is widely used in the existing literature [31]. In this task, participants were asked to assume the role of a charity staff member tasked with deciding which child to sponsor out of five options. Each child was described by five attributes presented in a hidden information matrix. Rows represented the five children (options), and columns represented five mutually conflicting attributes. To access information about each attribute, participants had to hover their mouse over a specific cell in the matrix, at which point the information would be revealed. Once the cursor moved to another cell, the previously displayed information was hidden again.

Participants were also randomly assigned to either a high-ETOD or low-ETOD condition. The manipulation was based on previous studies [31]. In the high-ETOD condition, participants were presented with extensive background information emphasizing the negative consequences of their decisions. For example, participants were told that children who were not selected may experience severe hardship, such as starvation. Additionally, they were shown pictures of malnourished children described as representatives of those supported by the charity. Participants in this condition were also asked to imagine that they had been supporting all five children previously and were now forced to reduce their support to just one child, leaving the other four unsupported and unlikely to receive help elsewhere. In contrast, participants in the low-ETOD condition were not presented with this background information. Instead, they were asked to imagine that they had not previously supported any children and were now choosing one child to sponsor for the first time. They were informed that the other four children were likely to receive support from other sources. All other aspects of the task were identical between the two groups.

As a manipulation check, participants completed the Self-Assessment Manikin (SAM) [58]. The SAM is a non-verbal pictorial measure of emotional responses across three dimensions: valence, arousal, and dominance. Given that the experimental manipulation specifically targeted emotional valence, only the valence subscale was utilized for the present analysis. Participants selected a figure from a continuum ranging from a smiling, happy figure (positive valence) to a frowning, unhappy figure (negative valence).

Participants then completed a probability weighting bias task designed to evaluate decision making under uncertainty. The task consisted of 72 experimental items generated by combining probability and monetary value information. To minimize participant burden, 14 loss scenarios were selected as experimental stimuli based on Pachur’s recommendations [5]. Each scenario presented participants with two options: a risky option, which offered a reward (*xi*) with probability (*pi*) or zero with probability (1 − *pi*), and a certain option, which provided a guaranteed reward (*zi*).

In low-probability loss scenarios, the expected utility of the risky option was slightly higher than that of the deterministic option (*piv(xi) > v(zi)*). Preference for the certain option under these conditions was interpreted as an overestimation of the probability of low-probability loss events. Conversely, in medium-to-high probability loss scenarios, the expected utility of the risky option was slightly lower than that of the deterministic option (*piv(xi) < v(zi)*). In such instances, selecting of the risky option indicated an underestimation of the probability of medium-to-high probability loss events. The total number of deviations from expected utility theory was calculated to measure the degree of probability weighting bias [5, 59, 60]. To reduce cognitive bias, six “double-risk” options were randomly inserted into the task, though these were excluded from the final analysis [61]. Additionally, all questions were randomized to prevent sequential effects. Counterbalancing was employed for question presentation format (vertical vs. horizontal), option presentation order (risky option first vs. certain option first), and information presentation sequence (probability first vs. amount first) among participants [61].

During the task, participants’ gaze times were recorded for both probabilistic and monetary information. A gaze time proportion was computed as the ratio of time spent fixating on probability cells to the total fixation time allocated to both probability and monetary cells. This proportion served as an indicator of participants’ decision-making strategies. A proportion below 0.5 indicated that participants focused more on monetary information and less on probability information, reflecting a non-compensatory cognitive strategy. Proportions close to 0.5 suggested comprehensive attention to both monetary and probability information, indicative of a compensatory cognitive strategy. Proportions above 0.5 implied greater focus on probability information and less attention to monetary information, again reflecting a non-compensatory cognitive strategy. These gaze time proportions were used to assess compensatory decision making during the task [61].

To account for potential confounding influences, the study also measured neurotic traits, as previous research indicates that neurotic traits are generally unaffected by event-related emotional distress but may influence decision-making tasks [62]. Finally, participants completed a demographic survey that collected information on age, gender, academic major, year of study, and place of residence.

### Data analyses

Statistical analyses were performed using the IBM SPSS and the PROCESS macro [63]. A 2 × 2 between-subjects design was employed, with induction (focused attention vs. mind-wandering) and ETOD (low vs. high) as independent variables. Descriptive statistics were calculated for all study variables. To assess the comparability of socio-demographic characteristics (e.g., age, gender, major, grade, and residence) and mental health variables (PHQ-2, GAD-2, neuroticism) across experimental conditions, 2 × 2 between-subjects ANOVAs were conducted for continuous variables, and binary logistic regressions were used for categorical variables. Manipulation check was performed to evaluate the effectiveness of the ETOD manipulation. The checks also utilized 2 × 2 between-subjects ANOVAs. The main dependent variables included processing time, probability weighting bias, and gaze time ratio. Separate 2 × 2 ANOVAs were conducted to examine the main effects of induction and ETOD, as well as their interaction, on each of these outcomes. Post-hoc simple effects analyses using Least Significant Difference (LSD) were conducted for significant interactions to explore differences between experimental conditions.

To examine whether attention allocation (gaze time ratio) mediates the relationship between ETOD and probability weighting bias and whether this process is moderated by induction, we conducted a moderated mediation analysis using PROCESS Model 5. This approach allowed for the assessment of the indirect effect of ETOD through gaze time ratio—utilizing 5,000 bootstrap samples for bias-corrected confidence intervals—while interaction terms were included to test for conditional effects based on the induction condition. For all analyses, *p* < .05 were considered statistically significant.

## Results

The final sample consisted of 138 participants, with no missing data. Participants were predominantly female (*n* = 116, 84.1%), with a mean age of 19.84 years (*SD* = 1.47) (see Table 1). The majority were third-year students (*n* = 66, 47.8%) enrolled in disciplines related to Arts and Humanities (*n* = 81, 58.7%) and residing in rural areas (*n* = 91, 65.9%).

To examine potential differences in socio-demographic characteristics across experimental conditions, 2 × 2 between-subjects ANOVAs were conducted for continuous variables, and logistic regressions were conducted for categorical variables. No significant differences were observed between conditions.

**Table 1:** Socio-demographic characteristics of the study participants (*n* = 138)

	Mind-wandering (*n* = 72)				Focused attention (*n* = 66)				*W*/*F*	*p*
	Low ETOD (*n* = 36)		High ETOD (*n* = 36)		Low ETOD (*n* = 34)		High ETOD (*n* = 32)			
	*M*/*n*	*SD*/%	*M*/*n*	*SD*/%	*M*/*n*	*SD*/%	*M*/*n*	*SD*/%		
Age	19.64	1.18	20.31	1.69	19.76	1.46	19.63	1.48	2.63	0.11
Gender									0.15	0.70
Female	31	86.1%	30	83.3%	28	82.4%	27	84.4%		
Male	5	13.9%	6	16.7%	6	17.6%	5	15.6%		
Major									3.05	0.08
Arts and Humanities	22	61.1%	23	63.9%	23	67.6%	13	40.6%		
Science	14	38.9%	13	36.1%	11	32.4%	19	59.4%		
Grade	2.42	0.97	2.81	1.04	2.41	1.05	2.31	1.03	1.97	0.16
Residence									0.18	0.67
Urban	8	22.2%	14	38.9%	11	32.4%	14	43.8%		
Rural	28	77.8%	22	61.1%	23	67.6%	18	56.3%		
PHQ-2	1.50	0.85	1.39	0.84	1.44	0.82	1.31	0.82	0.00	0.95
GAD-2	1.44	0.84	1.50	1.18	1.53	0.71	1.38	1.13	0.39	0.53
Neuroticism	45.47	7.57	44.69	9.56	47.12	6.92	45.22	7.21	0.17	0.68

To ensure that mental health and personality factors did not confound the experimental effects, group differences in depressive symptoms, anxiety, and neuroticism were examined. Mean PHQ-2 (*M* = 1.41, *SD* = 0.83) and GAD-2 (*M* = 1.46, *SD* = 0.98) scores indicated low levels of depression and anxiety in the sample, while the mean neuroticism score was 45.62 (*SD* = 7.88).

A series of 2 × 2 ANOVAs revealed no significant effects of induction, ETOD, or their interaction on depression, anxiety, or neuroticism, *F*s(1, 134) < 1.

As a manipulation check, the effectiveness of the ETOD was assessed using a 2 × 2 ANOVA on emotional valence (see Table 2). A significant main effect of ETOD was observed, *F*(1, 134) = 16.91, *p* < .001, *η*_*p*_² = 0.11, with participants in the low ETOD condition reporting more positive emotional valence (*M* = 3.77, *SE* = 0.12) compared to those in the high ETOD condition (*M* = 3.06, *SE* = 0.12, *p* < .001). However, the main effect of induction and the induction × ETOD interaction were not significant, *F*s(1, 134) < 1. These results demonstrate that ETOD, rather than induction, influenced participants’ emotional valence.

**Table 2:** Descriptive statistics of key experimental outcomes (*n*=138)

	Mind-wandering				Focused attention			
	Low ETOD		High ETOD		Low ETOD		High ETOD	
	*M*	*SD*	*M*	*SD*	*M*	*SD*	*M*	*SD*
Emotional Valence	3.75	0.97	3.03	0.94	3.79	1.122	3.09	1.02
Processing Time (in seconds)	84.43	28.53	144.65	67.81	89.91	44.06	107.03	42.17
Probability Weighting Bias	8.08	2.50	7.03	3.16	6.91	2.82	8.63	2.83
Gaze Time Ratio	0.47	0.03	0.46	0.03	0.47	0.02	0.46	0.02

The effects of induction and ETOD on processing time were examined using a 2 × 2 ANOVA. A significant interaction between induction and ETOD was observed, *F*(1, 134) = 6.94, *p* = .009, *η*_*p*_² = 0.05. Simple effects analysis revealed that under high ETOD conditions, participants in the mind-wandering condition required significantly longer processing times (*M* = 144.65, *SE* = 8.00) compared to those in the FA condition, *M* = 107.03, *SE* = 8.48, *F*(1, 134) = 10.41, *p* = .002. No significant differences were found between induction conditions under low ETOD, *p* > .05.

Probability weighting bias was also analyzed using a 2 × 2 ANOVA, revealing a significant interaction between induction and ETOD, *F*(1, 134) = 8.20, *p* = .005, *η*_*p*_² = 0.06. Contrary to expectations, pairwise comparisons showed that under high ETOD conditions, participants in the mind-wandering condition exhibited significantly lower probability weighting bias (*M* = 7.03, *SE* = 0.47) compared to those in the FA condition, *M* = 8.63, *SE* = 0.50, *F*(1,134) = 5.37, *p* = .02. No significant differences were observed between induction conditions under low ETOD, *p* > .05. Meanwhile, under the mind-wandering condition, no significant difference in PWB was observed across different levels of ETOD, *p* > .05.

Descriptive statistics and correlations for all study variables are presented in Table 3. As expected, emotional trade-off difficulties (ETOD) were significantly and negatively correlated with compensatory decision making (*r* = − .21, *p* < .05). Additionally, compensatory decision making was negatively correlated with probability weighting bias (*r* = − .21, *p* < .05).

**Table 3:** Descriptive statistics and correlations among study variables (*n* = 138)

	*M* ± *SD*	1	2	3	4
1 ETOD	1.49 ± 0.50	1			
2 CDM	0.47 ± 0.03	− 0.21^*^	1		
3 PWB	7.64 ± 2.89	0.05	− 0.21^*^	1	
4 Induction	1.48 ± 0.50	-0.02	-0.01	0.03	1

Note: CDM = Compensatory Decision Making. ^*^p< .05

**Fig. 1:**
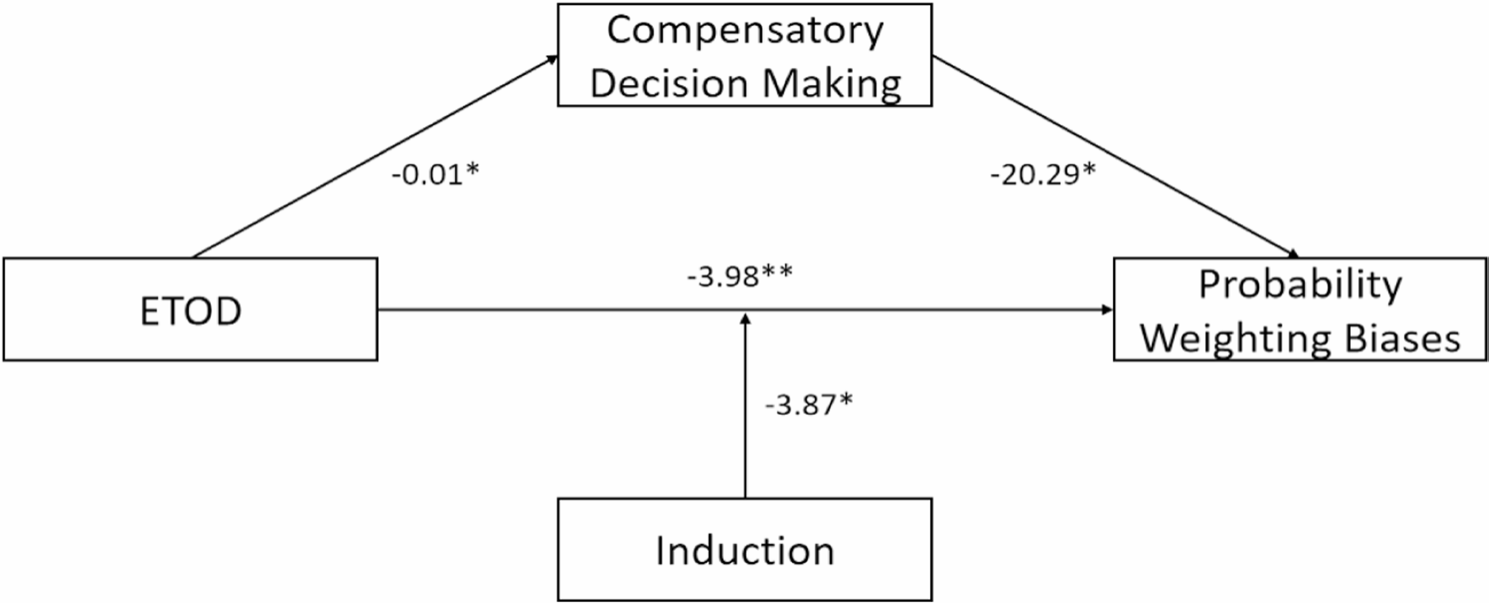
The moderated mediation model. *Note*: ^**^p < .01; ^*^p < .05

Finally, a moderated mediation analysis (PROCESS Model 5) was conducted to examine whether induction moderated the relationship between ETOD and probability weighting bias. Figure 1 illustrates the relationships between the variables in the model. The model was significant, *F*(4, 133) = 3.62, *p* = .008, *R*² = 0.10.

A significant interaction between ETOD and induction was found (*b* = 2.71, *p* = .005), indicating that induction moderated the relationship between ETOD and probability weighting bias. Specifically, in the FA condition, ETOD had a significant positive effect on probability weighting bias (*b* = 1.44, *SE* = 0.70, *p* = .04), with participants in the high ETOD condition exhibiting greater probability weighting bias compared to those in the low ETOD condition. In the mind-wandering condition, ETOD showed a marginally non-significant negative effect on probability weighting bias (*b* = -1.27, *SE* = 0.66, *p* = .06). The indirect effect was significant (indirect effect = 0.24, 95% *CI* [0.005, 0.61]). These findings suggest that ETOD primarily influences probability weighting bias through its impact on compensatory decision making. Table 4 presents the conditional indirect effects of ETOD on probability weighting bias moderated by induction.

**Table 4:** The moderated indirect effect

ETOD → Compensatory decision making → Probability weighting biases			
	Effect	*SE*	*LLCI*, *ULCI*
Induction			
Mind-wandering	-1.27	0.66	−2.58, 0.04
Focused attention	1.44	0.70	0.07, 2.82

## Discussion

The objectives of this study were threefold: (1) to investigate the impact of ETOD and induction on the PWF, (2) to examine whether compensatory decision making mediates the relationship between ETOD and the PWF, and (3) to explore whether induction moderates the effect of ETOD on the PWF. The findings, confirmed by a 2 × 2 ANOVA, reveal that under the control condition (mind-wandering), high ETOD requires significantly longer processing time but results in lower PWB. In contrast, under the FA condition, higher levels of ETOD lead to shorter processing time but higher PWB. Moreover, the results demonstrate that ETOD influences the PWF through compensatory decision making, while induction moderates the relationship between ETOD and the PWF. Specifically, in the FA condition, ETOD has a significant positive effect on PWB, whereas in the mind-wandering condition, ETOD exhibits a marginally non-significant negative effect on PWB.

The results partially support the notion that FA meditation can optimize decision-making outcomes and improve the PWF under the influence of ETOD. Specifically, under low ETOD conditions, focused attention appears to reduce PWB, resulting in more rational decision making. These findings are consistent with prior research [42], which suggests that FA meditation reduces the negative influence of task-induced emotions, such as those associated with decision-making trade-offs. Negative emotions from ETOD, which adversely affect the PWF and lead to more irrational decision making, may be regulated through FA meditation. We interpret this regulatory effect through the lens of our Attentional Regulation Perspective, grounded in Bishop et al.‘s two-component model [43]. According to the first component of this model, FA meditation explicitly trains top-down self-regulation of attention [12] —a process known to consume cognitive resources—to inhibit “elaborative processing”. It is important to note that the low ETOD condition consumes fewer cognitive resources than the high ETOD condition [30]. In this context, the attentional regulatory mechanism of FA meditation functions effectively to preserve the cognitive resources required for rational, compensatory processing, thereby mitigating PWB. This theoretical account is substantiated by neurological evidence regarding top-down control. Evidence indicates that mindfulness induction enhances activity in the left prefrontal cortex, a region critical for executive control and positive emotional states [64, 65]. Within our framework, this neural activation corresponds to the utilization of attentional resources to inhibit ETOD-induced elaborative processing. For instance, Creswell et al. found that mindfulness increased prefrontal cortex activation while concurrently reducing bilateral amygdala activity [66]. Since the amygdala is typically associated with negative emotional reactivity, its suppression by mindfulness through top-down regulation by the prefrontal cortex [67] serves as the neural substrate for the inhibition of ETOD-induced elaborative processing within the framework proposed by Bishop. Additionally, by enhancing activity in attention-related regions such as the anterior cingulate cortex, mindfulness facilitates a shift from automatic emotional responses to controlled attentional stability [68]. Consequently, under low ETOD, the resource investment required for top-down attentional regulation yields a net positive return. By successfully inhibiting the amygdala-driven elaborative processing, FA meditation preserves sufficient cognitive resources to support the complex trade-offs inherent in compensatory decision making. Complementing this, the second component of Bishop’s model (orientation to experience) also plays a critical role, as a non-judgmental attitude attenuates the impact of negative emotions associated with ETOD. Thus, the FA group could maintain rational decision standards by attenuating PWB, verifying the regulatory effect of FA meditation when the cognitive resource consumption induced by ETOD does not exceed the system’s regulatory capacity.

Notably, under high ETOD conditions, focused attention does not improve PWB; instead, it appears to exacerbate it. This finding stands in contrast to our initial hypothesis, which predicted that FA meditation would effectively attenuate decision quality decline across both high and low levels of ETOD. However, this divergence offers a crucial insight, reinforcing the core premise of our Attentional Regulation Perspective. From this perspective, the self-regulated attention behavior of the FA group in high ETOD situations is resource intensive. As evidenced by increased functional connectivity between frontoparietal and default mode networks observed in FA [12], this practice involves enhanced top-down regulation, marking it as an active process that inherently consumes cognitive resources. Unlike passive relaxation, the efficacy of this regulatory mechanism depends on a critical trade-off between resources conserved and resources invested. Crucially, the magnitude of this resource investment is determined by the practitioner’s proficiency. For novice meditators—such as those in this study—FA relies heavily on explicit, top-down executive processes, which consume significant cognitive resources [69]. Unlike experts who may regulate attention automatically, novices must exert continuous, effortful control to inhibit elaborative processing. Compared to low ETOD, the negative emotions generated by high ETOD impose a greater burden on cognitive resources. However, a more critical drain on resources arises from the heightened elaborative processing triggered by high ETOD, which functions as a persistent, high-intensity distracter. Consequently, this internal interference necessitates significantly more frequent and robust top-down inhibitory control to maintain attentional stability. For novice meditators, who rely on effortful rather than automated attentional control, this escalated need to suppress frequent intrusions creates a resource overload, where the cognitive cost of regulation exceeds the available cognitive resources. This net depletion forces the system to default to resource-sparing heuristic strategies—evidenced by the significantly shorter processing times observed in the FA group relative to the mind-wandering group—ultimately resulting in greater PWB. Our analysis yields two key insights regarding the mind-wandering condition. First, a direct test of H1—which predicted a positive effect of ETOD on PWB—was not supported. Second, under high ETOD conditions, the mind-wandering group exhibited significantly lower PWB compared to the FA group. These results further support the proposed Attentional Regulation Perspective. In contrast to the FA group, mind-wandering group does not necessitate the robust top-down attentional control typically required to inhibit heightened elaborative processing. We posit that mind-wandering inherently reduces self-awareness [42, 70], rendering it a passive state that requires minimal top-down attentional control. Unlike the active regulation in FA, mind-wandering avoids the high consumption of cognitive resources associated with attentional self-regulation. Under high ETOD, mind-wandering reduces the use of cognitive resources compared to FA, and inadvertently preserves remaining cognitive resources that the FA group exhausts on regulatory efforts. Consequently, while subject to the elaborative processing triggered by high ETOD, the mind-wandering group is spared the active attentional regulation required to inhibit it, thus retaining sufficient capacity to maintain a relatively better decision standard. This explains why the positive effect predicted by H1 was not observed in the mind-wandering condition and why “unrestricted mental wandering” (MW) paradoxically outperformed “active regulation” (FA) under conditions of extreme cognitive load.

It is worth noting that prior research has highlighted differences between task-related emotions (such as ETOD) and ambient mood. The negative effects of ambient mood tend to align with decision processing [27–30], typically resulting in more extensive, alternative-based processing. In contrast, the negative effects of task-related emotions often lead to inconsistencies in decision processing [31, 32], such as linking more extensive processing with attribute-based strategies. These differences may explain the conflicting outcomes of FA group observed in this study under varying levels of ETOD. Specifically, while FA meditation appears to improve decision making under low ETOD, its beneficial effects may be attenuated or reversed under high ETOD due to the significant cognitive resources required to regulate intense, task-related emotions. Future research should further delineate this boundary condition. For instance, if the emotional state is unrelated to the task (e.g., ambient mood), FA meditation may improve decision-making outcomes across all ETOD levels. However, this hypothesis requires empirical validation.

This study has several limitations that should be addressed in future research. Firstly, the negative emotions in this study were predicated on the assumption that the elicited emotional valence would be consistent across individuals. However, research has shown that emotions with different, or even similar, valences can influence decision making in varying ways [71]. Future studies should explore the specific types of emotions triggered by ETOD and their differential effects on decision-making processes. Secondly, the sample is almost entirely female (approximately 84.1%), which may affect the generalizability of the results. Suggest future research to balance the gender ratio. Thirdly, as this study relied on a brief induction and behavioral data, we could not directly observe the neural mechanisms or temporal dynamics of resource allocation. Given that short-term interventions may differ from long-term practice [18, 72], future studies should use objective physiological measures (e.g., EEG, fMRI) and long-term protocols to validate the specific neural processes involved in high ETOD contexts. Fourthly, while we implemented procedural safeguards to ensure basic understanding of the induction instructions, the study lacked a quantitative participant-level manipulation check. Finally, we must acknowledge a methodological limitation regarding the verification of the induction. We excluded the MAAS data to ensure rigor, as this dispositional measure is methodologically unsuitable for detecting state changes following a brief 11-minute induction. Consequently, without a direct manipulation check, we cannot definitively confirm that the observed effects were driven specifically by the intended state of FA meditation rather than other factors. Despite this limitation, the validity of the FA meditation manipulation in this study is supported by several considerations. First, although the results from the previous MAAS measurement reached statistical significance, the effect size was small. The presence of this small effect is precisely because trait mindfulness itself requires repeated practice to improve [73], and the small effect size reflects the outcome of a single practice session. Second, although the effect size we identified was very small, we observed significant group differences in outcomes. Crucially, our preliminary analysis confirmed that there were no significant differences between the two groups in terms of sociodemographic characteristics (e.g., age, gender) or baseline psychological traits (e.g., PHQ-2, GAD-2, and Neuroticism). Given this established group equivalence, the observed divergence in outcomes implies inherent differences between the two conditions that are likely attributable to the mindfulness manipulation itself [55]. Finally, there are inherent limitations in using self-reported measures as a manipulation check for mindfulness [74]. Future research should employ state-specific measures or objective physiological indicators (e.g., EEG or HRV) to rigorously validate the induction efficacy. Interestingly, under high ETOD conditions, this study provides evidence suggesting that the additional cognitive resource consumption generated by the top-down emotion regulation mechanisms of mindfulness can lead to reliance on heuristic strategies, which are manifestations of decision avoidance. This finding highlights the potential limitations of brief mindfulness inductions in high-stress decision making contexts, particularly for novice practitioners who may struggle with resource depletion during FA tasks.

## Conclusions

Despite these limitations, this study confirmed the partial improvement effect of FA meditation on decision making, particularly under low ETOD conditions. However, the limited impact observed at high ETOD levels elucidates the boundaries of attentional regulation. Given that FA is an active process requiring cognitive resources, our findings suggest that high ETOD conditions overwhelm this capacity. Thus, FA effectiveness is strictly context-dependent, restricted to situations where the cognitive cost of self-regulation does not overwhelm the system’s available resources.

## Data Availability

The datasets generated and analyzed during the current study will be available from the author upon reasonable request.

## Abbreviations

PWB: probability weighting bias
PWF: probability weighting function
ETOD: Emotional trade-off difficulties
